# Buprenorphine alters microglia and astrocytes acutely following diffuse traumatic brain injury

**DOI:** 10.1038/s41598-021-88030-z

**Published:** 2021-04-21

**Authors:** Jane Ryu, Phillip Stone, Sabrina Lee, Brighton Payne, Karen Gorse, Audrey Lafrenaye

**Affiliations:** 1 Virginia Commonwealth University, 1101 E. Marshall St., Box 980709, Richmond, VA 23298 USA; 2 Godwin High School, Henrico, VA USA; 3 University of Mary Washington, Fredericksburg, VA USA

**Keywords:** Neuroimmunology, Diseases of the nervous system, Astrocyte, Microglia, Oligodendrocyte, Myelin biology and repair, Neuroscience, Cellular neuroscience

## Abstract

Traumatic brain injury (TBI) is a common phenomenon, accounting for significant cost and adverse health effects. While there is information about focal pathologies following TBI, knowledge of more diffuse processes is lacking, particularly regarding how analgesics affect this pathology. As buprenorphine is the most commonly used analgesic in experimental TBI models, this study investigated the acute effects of the opioid analgesic buprenorphine (Bup-SR-Lab) on diffuse neuronal/glial pathology, neuroinflammation, cell damage, and systemic physiology. We utilized a model of central fluid percussion injury (CFPI) in adult male rats treated with a single subcutaneous bolus of Bup-SR-Lab or saline 15 min post-injury. Microscopic assessments were performed at 1 day post-injury. Cell impermeable dextran was infused intraventricularly prior to sacrifice to assess neuronal membrane disruption. Axonal injury was assessed by investigating labeling of the anterogradely transported amyloid precursor protein. Neuroinflammation was assessed by analyzing Iba-1 + microglial and GFAP + astrocyte histological/morphological features as well as cytokine levels in both regions of interest (ROIs). Myelin pathology was assessed by evaluating the expression of myelin basic protein (MBP) and the propensity of MBP + myelin debris. Acute physiologic data showed no difference between groups except for reduction in weight loss following cFPI in Bup treated animals compared to saline. There were no discernable differences in axonal injury or membrane disruption between treatment groups. Cytokine levels were consistent between Bup and saline treated animals, however, microglia and astrocytes revealed region specific histological changes at 1d following Bup treatment. Myelin integrity and overall MBP expression showed no differences between Bup and saline treated animals, but there were significant regional differences in MBP expression between the cortex and thalamus. These data suggest effects of Bup treatment on weight following CFPI and potential regional specificity of Bup-associated microglial and astrocyte alterations, but very little change in other acute pathology at 1-day post-injury. Overall, this preliminary study indicates that use of Bup-SR-Lab in preclinical work does have effects on acute glial pathology, however, longer term studies will be needed to assess potential effects of Bup treatment on more chronic pathological progressions.

## Introduction

Traumatic brain injury (TBI) is an increasingly common phenomenon, with almost 2 million reported cases occurring annually in the US alone^1–3^. Overall, TBIs contribute to nearly 30% of all injury-related deaths, and account for significant healthcare cost and adverse health effects. Brain injury-induced pathology can be subclassified into focal and diffuse changes. Focal pathology of TBI results from the impact of the brain, and results in relatively homogenous sequelae of injury. Diffuse pathology, on the other hand, can be widely distributed throughout the brain in “pockets” of injury, and involves heterogenous cellular responses, ranging from diffuse axonal injury (DAI) and neuronal membrane disruption to neuroinflammatory changes and myelin pathology, making it difficult to track in the human population. Therefore, animal models of TBI are utilized for the rigorous assessments of TBI-induced pathology. These experimental TBI studies are being brought into greater alignment modeling the common data elements utilized in clinical TBI studies by groups such as the Federal Interagency Traumatic Brain Injury Research (FITBIR) data sharing group^4^ making metanalysis of experimental studies much more feasible.

As various models of experimental diffuse TBI require surgical intervention, guidelines suggest the use of analgesics^5,6^. The most commonly used analgesics in experimental studies are opioids. However, there are indications that opioids may alter neuronal survival and possibly inflammation^7–11^, and the effects of opioids on various other pathologies have not been rigorously tested. Because effects of analgesics on neuropathology and physiology are poorly described, it is unclear if their use confounds data. This uncertainty regarding possible secondary effects of opioid administration post-injury has led to a debate regarding the use of analgesics following TBI.

Buprenorphine (Bup) is a semi-synthetic opioid derived from thebaine, one of six naturally occurring alkaloids of the opium poppy^10,12^. It is a partial agonist of the Mu receptor and an antagonist of the Kappa and Delta opioid receptors^12,13^. Due to these binding properties, Bup is commonly used in the treatment of opioid use disorder, the context in which the drug is most often studied^14,15^. Bup is also the most commonly used analgesic in pre-clinical animal models owing to the effectiveness of the sustained release formulation of Bup, Bup-SR-lab, in pain reduction over multiple days following a single subcutaneous administration^16–21^. While there are indications that Bup could affect cellular pathology^7,22,23^, the specific effects of Bup-SR-Lab on neuronal/glial pathology, neuroinflammation, cell damage, and physiology following brain injury are still unclear. Therefore, this study sought to determine the effects of Bup-SR-Lab on various acute diffuse pathologies precipitated by TBI. Specifically, physiological changes, neuronal membrane disruption, axonal injury, microglial and astrocyte alterations, cytokine expression and myelin changes were assessed at 1 day following diffuse central fluid percussion injury (CFPI) in adult male Sprague–Dawley rats treated subcutaneously with the veterinarian and pharmaceutical company recommended dose of 1 mg/kg Bup-SR-Lab or saline at 15 min post-injury.

## Methods

### Animals

Experiments were conducted in accordance with ARRIVE guidelines^24^ and the Virginia Commonwealth University institutional ethical guidelines concerning the care and use of laboratory animals and were approved by the Institutional Animal Care and Use Committee at Virginia Commonwealth University, which adhere to regulations including, but not limited to, those set forth in the “Guide for the Care and Use of Laboratory Animals: 8th Edition” (National Research Council). Overall, 12 adult (12 to 16-week-old; n = 6/group) male Sprague–Dawley rats were used for this study. Animals were housed in individual cages on a 12-h light–dark cycle with free access to food and water and full veterinary oversight.

### Surgical preparation, injury induction, and drug administration

Anesthesia was induced with 4% isoflurane in 30% O_2_/70% room air. Animals were then intubated and ventilated with 1.5–2.5% isoflurane in 30% O_2_ and 70% room air throughout the duration of the surgery, injury, and physiologic monitoring. Body temperature was maintained at 37 °C with a rectal thermometer connected to a feedback-controlled heating pad (Harvard Apparatus, Holliston, MA, USA). All animals were placed in a stereotaxic frame (David Kopf Instruments, Tujunga, CA, USA). A midline incision was made followed by a 4.8 mm diameter circular craniectomy, which was positioned along the sagittal suture midway between bregma and lambda. The dura was left intact. A 2 mm diameter burr hole was also drilled into the left parietal bone overlaying the left lateral ventricle (0.8 mm posterior, 1.3 mm lateral, and 2.5 to 3 mm ventral relative to bregma) through which a 25-gauge needle, connected to a pressure transducer and a micro infusion pump 11 Elite syringe pump (Harvard Apparatus) via PE50 tubing, was placed into the left ventricle. Appropriate placement of the infusion pump into the lateral ventricle was verified via a 2.3 μl/min infusion of sterile saline within the closed fluid pressure system during needle placement^25,26^. The needle was held in the lateral ventricle for at least 5 min to record preinjury ICP; then the needle was slowly removed. Bone wax was used to seal the burr hole used for the ICP measurements before preparation for central fluid percussion injury (CFPI). The procedures used to induce CFPI were consistent with those described previously^25–28^. Briefly, a Luer-Loc syringe hub was affixed to the craniotomy site with dental acrylic (methyl methacrylate; Hygenic, Akron, OH, USA) that was applied around the hub, including the area overlying the sealed burr hole and allowed to harden. Animals were removed from the stereotaxic frame and placed on a raised platform for connection to the fluid percussion device, maintaining an unbroken fluid-filled system from the intact dura through the cylinder, via a Leur-Loc adaptor. During injury the investigator supported the animal’s body on the platform but did not hold the head allowing the Leur-Loc mechanism to maintain connection between the injury hub and fluid percussion device. To induce a mild-moderate CFPI a pendulum was released onto the fluid-filled cylinder of the FPI device, producing a pressure pulse of 2.05 ± 0.10 atmospheres for ~ 22.5 ms (Table 1), which was transduced through the intact dura to the CSF. The pressure pulse was measured by a transducer affixed to the injury device and displayed on an oscilloscope (Tektronix, Beaverton, OR, USA). Immediately after the injury, animals were reconnected to the ventilator and physiologic monitoring devices. The hub, dental acrylic, and bone wax were removed *en bloc* and Gelfoam was placed over the craniectomy/injury site. The animal was then replaced in the stereotaxic frame, and the ICP probe was reinserted into the lateral ventricle, as described above, for postinjury ICP monitoring. Immediate post-injury physiology was recorded for 15 min after CFPI followed by subcutaneous administration of either 1 mg/kg Bup SR-Lab or saline. The surgeon randomly selected a pre-filled blinded syringe that was administered by another investigator to avoid inadvertent unblinding of the surgeon due to the difference in viscosity of the solutions that might introduce bias that could influence animal care. One hour following injury the scalp was sutured and treated with lidocaine and triple-antibiotic ointment. Rats were then allowed to recover and were returned to clean home cages.

**Table 1:** Physiological readouts in buprenorphine and saline treated animals prior to and following cFPI.

	Saline	Bup
Pre-injury Weight (g)	509 (87)	545 (61)
Injury Intensity (atm)	2.05 (0.08)	2.01 (0.10)
Injury duration (msec)	22.34 (0.64)	22.37(0.21)
Recovery Time (min)	19.6 (7.4)	79.9 (78.9)
Δ ICP 10 min-1d	8.51 (6.74)	7.51 (4.04)

g, grams; atm, atmospheric pressure; BPM, beats per minute; **Δ**ICP 10 min-1d, change in intracranial pressure from 10 min post-injury to 1d post-injury. n = 6 rats/group. Data presented as mean (standard deviation).

### Physiologic assessment

**Figure 1:**
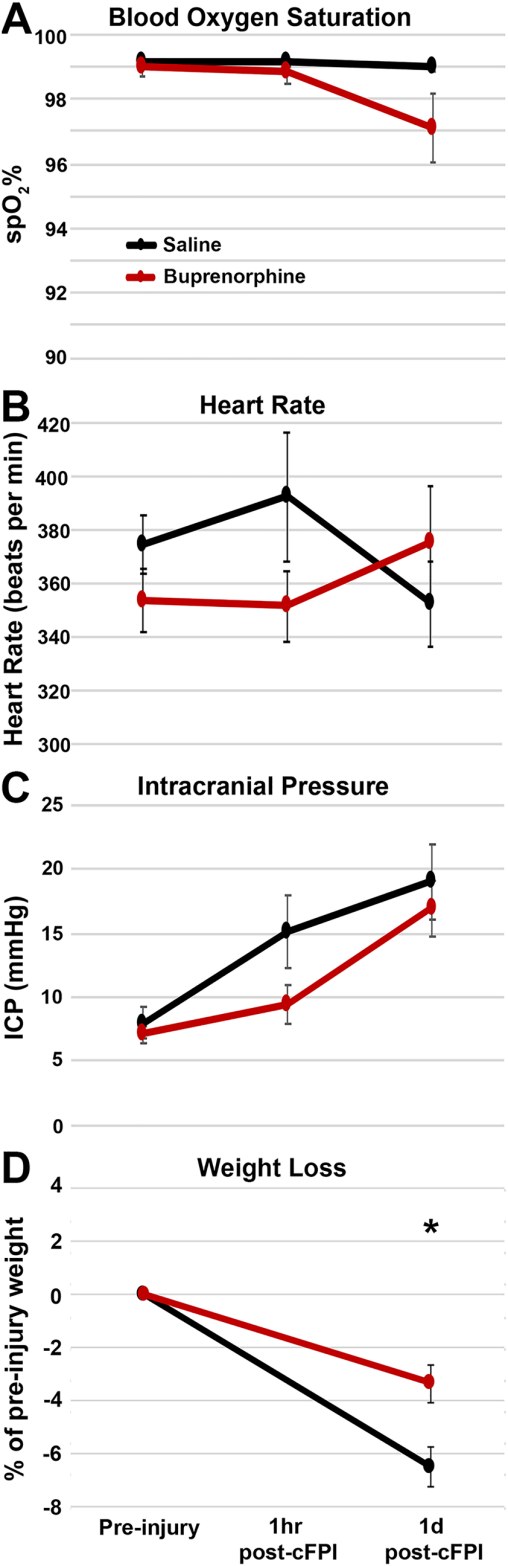
Weight loss was reduced in animals treated with Bup-SR-Lab at 1-day post-injury. Physiological readouts in saline and buprenorphine (Bup) treated adult male rats following central fluid percussion injury (cFPI). (**A**) Blood oxygen saturation, (**B**) heart rate, and (**C**) Intracranial pressure were unchanged with Bup. (**D**) Weight loss 1d following cFPI was less substantial in cFPI rats treated with Bup compared to saline. Figure was compiled using Adobe Photoshop CS., version 22.0 (2020), San Diego, CA. n = 6 rats/group. Mean ± SEM. *p < 0.05 compared to saline.

Heart rate, respiratory rate, and hemoglobin oxygen saturation were monitored via a hindpaw pulse oximetry sensor (STARR Life Sciences, Oakmont, PA, USA) for the duration of anesthesia, except during the induction of injury. Intracranial pressure (ICP) was measured intraventricularly, as described above. All physiologic measurements were recorded using a PowerLab System (AD Instruments, Colorado Springs, CO, USA). All animals maintained systemic physiological homeostasis throughout the experiment (i.e., heart rate > 200 beats per min and oxygenation > 90%; Table 1; Fig. 1). Changes in ICP following CFPI were noted in both groups, particularly at 1d post-injury (Table 1; Fig. 1C). Recovery time following CFPI (the time from withdrawal of inhaled anesthetic to first movement) and weight loss (percent reduction in animal weight from pre-injury to 1d post-injury) were also assessed (Table 1; Fig. 1).

### Tracer infusion

At 1d post-injury rats were anesthetized with 4% isoflurane in 30% O_2_/70% room air followed by maintenance dose of 2% isoflurane in 30% O_2_/70% room air via a nose cone. Animals were secured into a stereotaxic device and the incision from the previous day was reopened by removing the sutures. The ICP needle was filled with 0.7 mg/17 μl 10 kDa biotinylated dextran and placed into the left lateral ventricle with continuous ICP monitoring, as described above. The ICP at 1d post-CFPI was measured for 15 min following needle placement then the dextran was infused at a rate of 1.3 μl/min with continuous ICP monitoring. The tracer was allowed to diffuse for 2 h before animals underwent transcardial perfusion, as described below.

### Tissue processing

At 1d post-CFPI 2 h following tracer infusion, anesthetized rats were overdosed with Euthasol euthanasia-III solution (Henry Schein, Dublin, OH, USA) followed by transcardial perfusion with cold 0.9% saline. As was described previously^26^, both fresh and fixed brain tissue was collected from each animal. A tissue core of the right lateral neocortex and thalamus/midbrain was taken for molecular assessments prior to transcardial fixation with 4% paraformaldehyde/0.2% glutaraldehyde in Millonig’s buffer (136 mmol/L sodium phosphate monobasic/109 mmol/L sodium hydroxide) for immunohistochemical analysis of the left side of the brain. After transcardial perfusion, the left side of the brain was removed and postfixed for > 72 h. Postfixed brains were sectioned coronally in 0.1 mmol/L phosphate buffer with a vibratome (Leica, Banockburn, IL, USA) at a thickness of 40 μm from bregma to ∼ 4.0 mm posterior to bregma. Sections were collected serially in 12-well plates and stored in MIllonig’s buffer at 4 °C. All quantitative analyses were performed at least 1 mm posterior to the needle track used for ICP monitoring. The well from which sections would be taken for analysis was selected via a random number generator (1–12) and the first 4 sections with visible hippocampus were taken representing serial sections throughout the rostral-caudal extent (1.8 mm ± 0.2 mm to 3.8 mm ± 0.2 mm posterior to bregma, each 480 μm apart). Histologic analyses were performed on the left lateral somatosensory cortex restricted to layers V and VI extending from the area lateral to CA1 to the area lateral to CA3 of the hippocampus and entire left hemi-thalamus extending from the midline and dorsal surface of the thalamus to the reticular nucleus and zona inserta of the thalamus laterally and ventrally.

### Assessment of cell damage

To evaluate the numbers of damaged cells in the cortex and thalamus of rats following injury and vehicle or Bup treatment, four sequential, randomly selected sections per animal were stained with hematoxylin and eosin (H&E) and assessed as described previously^25,26,29^. Briefly, tissue was mounted on gelatin-coated slides before dehydration and rehydration. Rehydrated tissue was incubated in Gills hematoxylin (Leica Biosystems, Buffalo Grove, IL, USA) followed by bluing agent (Leica Biosystems) and three dips in 0.25% eosin Y/0.005% acetic acid/95% ethanol before sections were cleared through increasing concentrations of ethanol and cover-slipped with Permount (Thermo Fisher Scientific, Waltham, MA, USA). Sections were visualized using a Nikon Eclipse 800 microscope. Assessments were done on the left side of the cortex and thalamus for each section. The number of damaged neurons, delineated by eosinophilic cytoplasm and condensed nuclei, in the entire left lateral neocortex and thalamus was counted by two independent investigators blinded to the animal group and averaged for each animal and each group. Data is reported as the number of damaged cells/region of interest (ROI).

### Assessment of axonal injury

To quantify axonal injury, immunohistochemistry targeting amyloid precursor protein (APP) was performed. Sections were immunolabeled, as previously described^25,30^. Tissue was blocked and permeabilized in 5% normal goat serum and 1.5% triton followed by overnight incubation with a primary rabbit antibody against the C terminus of *β*-APP (Cat. #51-2700, 1:700, Life Technologies). Secondary antibody, biotinylated goat anti-rabbit IgG (Cat. #BA-1000, 1:1000, Vector Laboratories, Burlingame, CA, USA) was then incubated for 2 h at room temperature. The sections were subsequently incubated in avidin biotinylated enzyme complex using the Vectastain ABC kit (Vector Laboratories) followed by visualization with 0.05% diaminobenzidine/0.01% H_2_0_2_/0.3% imidazole/phosphate-buffered saline. The tissue was mounted, dehydrated, and cover-slipped. Visualization of APP-labeled axonal swellings was performed using a Nikon Eclipse 800 microscope (Nikon, Tokyo, Japan) equipped with an Olympus DP71 camera (Olympus, Center Valley, PA, USA). The total number of APP + axonal swellings in the entire region of interest (the left lateral neocortex layers V and VI or the left thalamus) for each section was counted by eye by an investigator blinded to animal group.

### Immunofluorescence

To identify microglia, astrocytes, and myelin, fluorescent immunohistochemistry against the calcium binding protein, Iba-1 (microglia), glial fibrillary acidic protein, GFAP (astrocytes), or myelin basic protein, MBP (myelin) was done. Briefly, 40 μm thick coronal sections were blocked and permeabilized in 1.5% triton and 5% normal goat serum followed by overnight incubation with primary antibody rabbit anti-Iba-1 (Cat. #019-19741, 1:1000, Wako; Osaka, Japan), mouse anti-GFAP (Cat.#MAB3402, 1:1000; GA5, Millipore, Burlington, MA, USA) or mouse anti-myelin basic protein (Cat #808401; 1:1000; SMI99, BioLegend, San Diego, CA, USA) overnight followed by incubation with Alexa Fluor 488-conjugated goat anti-rabbit secondary antibody (Cat.# A11034, 1:700; ThermoFisher Scientific) or Alexa Fluor 568-conjugated goat anti-mouse secondary antibody (Cat.# A-11031, 1:700; ThermoFisher Scientific). Tissue was mounted using Vectashield hardset mounting medium with Dapi (Cat.#H-1500; Vector Laboratories). Immunolabeling for all tissue was done at the same time to reduce run-to-run variability. All image acquisition settings were held consistent between groups for each region of interest (left lateral neocortex layers V and VI or the left thalamus) and imaging was done by an investigator blinded to animal group. Dapi nuclear labeling was used to verify focus and restriction within the regions of interest prior to image acquisition.

### Assessment of neuronal membrane disruption

Consistent with previous studies, we assessed the potential for neuronal membrane disruption via the utilization of tagged 10 kDa dextran^25,26,31^, which are impermeable to cells with intact membranes. Cells containing dextran, therefore, indicate membrane disruption. Immunolabeling for all tissue was done at the same time to reduce run-to-run variability. Sections were blocked with 5% normal goat serum, permeabilized with 1.5% triton, and immunolabeled with primary antibodies mouse anti-NeuN (Cat. #MAB377, 1:500; A60, Millipore), to identify neurons, and goat anti-biotin (Cat #31852, 1:2,000, ThermoFisher Scientific; Waltham, MA, USA) to identify dextran. Secondary antibodies Alexa-fluor 568-conjugated donkey anti-goat IgG (Cat. #A11057, 1:700, ThermoFisher Scientific) and Alexa-fluor 488-conjugated goat anti-mouse IgG (Cat. #A11001, 1:700, ThermoFisher Scientific) were then incubated and the tissue was mounted with Vectashield hardset mounting medium with DAPI (Cat.#H-1500; Vector Laboratories). Sections were imaged by confocal microscopy using a Zeiss LSM 700 System (Carol Zeiss, Oberkochen, Germany). Confocal images of the left neocortex layers V and VI were taken at × 40 magnification in a systematically random fashion by an investigator blinded to animal group using DAPI labeling to verify focus and NeuN label to verify location within the region of interest. Image acquisition settings were held constant for comparable regions for all groups analyzed. Analysis of NeuN^+^ neurons containing the cell-impermeable dextran was performed by an investigator blinded to animal group using the ImageJ colocalization finder plugin and traditional cell counting. Dextran containing neurons were quantified for each image and averaged for each animal.

### Assessment of microglial activation

Four sections/animal labeled with Iba-1 were imaged using an Olympus DP71 camera (Olympus, Center Valley, PA, USA) and were analyzed using FIJI/ImageJ as follows. All cells in which both the cell body and process network were in focus were marked in each image and a subset (n = 5/image) were randomly selected using a random number generator for further morphological analysis. Each randomly selected cell was individually analyzed for process number, number of branch end/terminal points, and maximum process segment length using the skeleton analysis tool in ImageJ. A complexity index was also calculated for each microglia using the formula, complexity index = number of processes/number of end points, with a lower number indicating reduced process complexity. The soma of each cells was also circumscribed to assess the perimeter of the cell body through ImageJ particle analysis. All data was recorded by an investigator blinded to animal group and averaged for each image. Individual microglia were considered ns.

### Assessment of astrocyte activation

Three randomly selected images in a randomly selected section were taken at 20 × magnification under consistent microscope settings for each region of interest (left lateral neocortex layers V and VI or the left thalamus) using a Keyence BZ-X800 microscope with section scanning on to reduce background (Keyence Corporation of America, Itasca, IL, USA). Images were processed with background subtraction and automatic thresholding to generate masks of GFAP + astrocytes. All cells within the mask were added to the Region of Interest Manager in FIJI/ImageJ. Measurements of the number of astrocytes/image, cellular area and circularity of individual astrocytes and the percent of GFAP + astrocyte coverage/image were assessed. All data was analyzed by an investigator blinded to animal group and averaged for each image and each animal. Individual animals were considered ns.

### Assessment of myelin integrity

Four sections per animal with 6 images/section for the cortex or 4 images/section for the hemi-thalamus were imaged at 40 × magnification in a systematically random fashion for each ROI using a Keyence BZ-X800 microscope with section scanning on to reduce background (Keyence Corporation of America). All images were captures and analyzed by an investigator blinded to animal group using the DAPI label to verify focus and location within the ROI. Image acquisition settings were held constant for comparable regions (cortex or thalamus) for all groups analyzed. Analysis of intact myelin fibers and myelin debris was performed using the Analyze particle plugin in FIJI/ImageJ (National Institutes of Health) with size and circularity parameters for object differentiation. The parameters used to determine myelin debris were circularity = 0.3–1.0 and particle size = 0.5–10 µm^2^. To assess myelin fibers the analysis settings were as follows, circularity = 0.0–0.1 and particle size = 25-infinity µm^2^. The average total area covered by intact myelin fibers or myelin debris was quantified for each image and averaged for each animal. Individual animals were considered ns.

### Quantification of protein expression

Tissue from the right lateral neocortex and thalamus was homogenized in NP40 Buffer (150 mM NaCl, 50 mM Tris pH 8.0, 1% Triton-X) and protease inhibitor cocktail (AEBSF 10.4 mM, Aprotinin 8 μM, Bestatin 400 μM, E-64 140 μM, Leupeptin 8 μM, Pepstatin A 150 μM, Cat#: P8340, Sigma, Saint Louis, MO, USA). Protein concentration was determined using a bicinchoninic acid assay in accordance with manufacturer’s instructions (Cat#23225; ThermoFisher) and quantified on a PHERAstar Spectrophotometer (BMG Labtech, Cary, NC, USA).

For assessment of cytokine expression, cortical or thalamic protein homogenates were sent to Quansys Bioscience (Logan, UT, USA) for cytokine analysis of rat IL-1a, IL-1b, IL-2, IL-4, IL-6, IL-10, IL-12, IFNy, and TNFa. Three replicates were run for each sample and the means of the replicates were used for each animal. Cytokine concentration (in pg) was normalized to total protein concentrations (in mg) for each sample. Samples in which there was no detectable amount of cytokine were set to 0 pg/mg for analysis (IL-1b Thalamus saline n = 3, Thalamus Bup n = 1; IL-12 Thalamus saline n = 4, Thalamus Bup n = 4) after verifying acceptable (> 0.5 mg/ml) total protein concentrations.

To analyze myelin basic protein (MBP) expression, Western blotting was performed. Protein (15 ug) was boiled for 10 min in 2 × Laemelli loading buffer and run at 200 V for 30 min on Mini-PROTEAN TGX Stain-free 4–20% precast polyacrylamide gels (Cat #4,568,096; BioRad, Hercules, CA, USA). Protein was transferred onto 0.2 um PVDF membranes using a Transblot Turbo transfer system (Bio-Rad) under the low molecular weight manufacturer settings (2.5 Amps, 25 V for 5 min). Western blotting was done on an iBind flex apparatus (Invitrogen) using primary antibodies rat anti-myelin basic protein (1:1000, Cat #MAB386; Millipore Sigma) and mouse anti-actin (1:4000, Cat #66009-1-Ig; Proteintech; Rosemont, IL, USA) followed by anti-rat-HRP secondary antibody (1:5000; Cat#112-035-003; Jackson Laboratories, West Grove, PA) and anti-mouse-HRP secondary antibody (1:5000, Cat #115-035-003; Jackson Laboratories; West Grove, PA). Chemiluminescent images were taken on a ChemiDoc imaging system (BioRad). Densitometric analysis was done in ImageJ (National Institutes of Health) for actin and total MBP expression, as well as individual MBP isoform expression. Total MBP expression was measured by taking the densities of each individual MBP isoform band and adding them to get the total sum. This method reduced biasing of the analysis by the variable degrees of white space between the MBP isoform bands in each run. MBP was then normalized to actin and to sham controls. All western blots were run in triplicates on three separate gels to reduce run-to-run variability potentially biasing the results.

### Statistical analysis

A Shapiro–Wilk test for normality of the data was done prior to statistical analysis. The number of animals to be assessed for each group was determined by power analysis using previous data, an alpha = 0.05 and a power of 80%. One-way or two-way ANOVA were done with Bonferroni post-hoc corrections for multiple comparisons. Statistical significance was set to a *p* value < 0.05. Data are presented as mean ± standard error of the mean (SEM) unless otherwise indicated.

### Ethics approval

Experiments were conducted in accordance with the Virginia Commonwealth University institutional ethical guidelines concerning the care and use of laboratory animals (Institutional Animal Care and Use Committee, Virginia Commonwealth University), which adhere to regulations including, but not limited to, those set forth in the “Guide for the Care and Use of Laboratory Animals: 8th Edition” (National Research Council).

## Results

### Post-injury weight loss was decreased with Bup-SR-Lab treatment

As has been well characterized previously, CFPI (2.05 ± 0.1 atmospheres) did not generate gross tissue pathology^25,26,28,29^. While there was apparent sub-arachnoid bleeding at 1d post-injury, there was no apparent damage to the underlying cortex. Neither saline nor Bup treated animals demonstrated indications of contusion, hematoma formation, or overt cortical compression.

Body temperature was maintained at 37 °C with a feedback loop thermoregulatory system connected to a rectal thermometer to avoid potential confounds of hypothermia in either group. As drug treatment was both randomized and blinded there was little possibility to fully match injury metrics between treatment groups, however, there was no difference in pre-injury weight (One-way ANOVA F_1,12_ = 0.73, *p* = 0.411), injury intensity (One-way ANOVA F_1,12_ = 0.68, *p* = 0.427), or injury duration (One-way ANOVA F_1,12_ = 0.013, *p* = 0.913) between groups (Table 1). While recovery time (time from withdrawal from anesthesia to first sporadic movements) was higher in the Bup treated group on average, there was substantial variability in this group and ultimately this difference was not significant (Table 1; One-way ANOVA F_1,12_ = 2.82, *p* = 0.124).

To evaluate the potential effects of Bup on physiology, blood oxygen saturation, heart rate, and intracranial pressure were assessed prior to injury and at 1 h and 1d following CFPI. Animal weight was also assessed prior to and 1d following CFPI to explore potential effects of Bup on post-injury weight loss. There was no discernable difference in blood oxygenation or heart rate between saline and Bup treated animals at any time point assessed (Fig. 1A,B). Intracranial pressure was also comparable between treatment groups at all time points measured (Fig. 1C; Two-Way ANOVA F_1,22_ = 2.37; *p* = 0.138). A significant increase in intracranial pressure of about 8 mmHg from 1 h to 1d post-injury was, however, noted in both treatment groups (Two-Way ANOVA F_1,22_ = 5.50; *p* = 0.028). This change in intracranial pressure was also consistent between saline and Bup treated groups (Table 1). Weight loss, however, was significantly reduced in the Bup treated group (3.36 ± 0.72% loss from pre-injury weight) as compared to the saline treated group (6.53 ± 0.78% loss from pre-injury weight; One-way ANOVA F_1,11_ = 8.81; *p* = 0.013). This difference in weight loss was not correlated to pre-injury weight (spearman Rho = 0.14, *p* = 0.648).

### Treatment with Bup-SR-Lab did not alter neuronal somatic or axonal injuries

**Figure 2:**
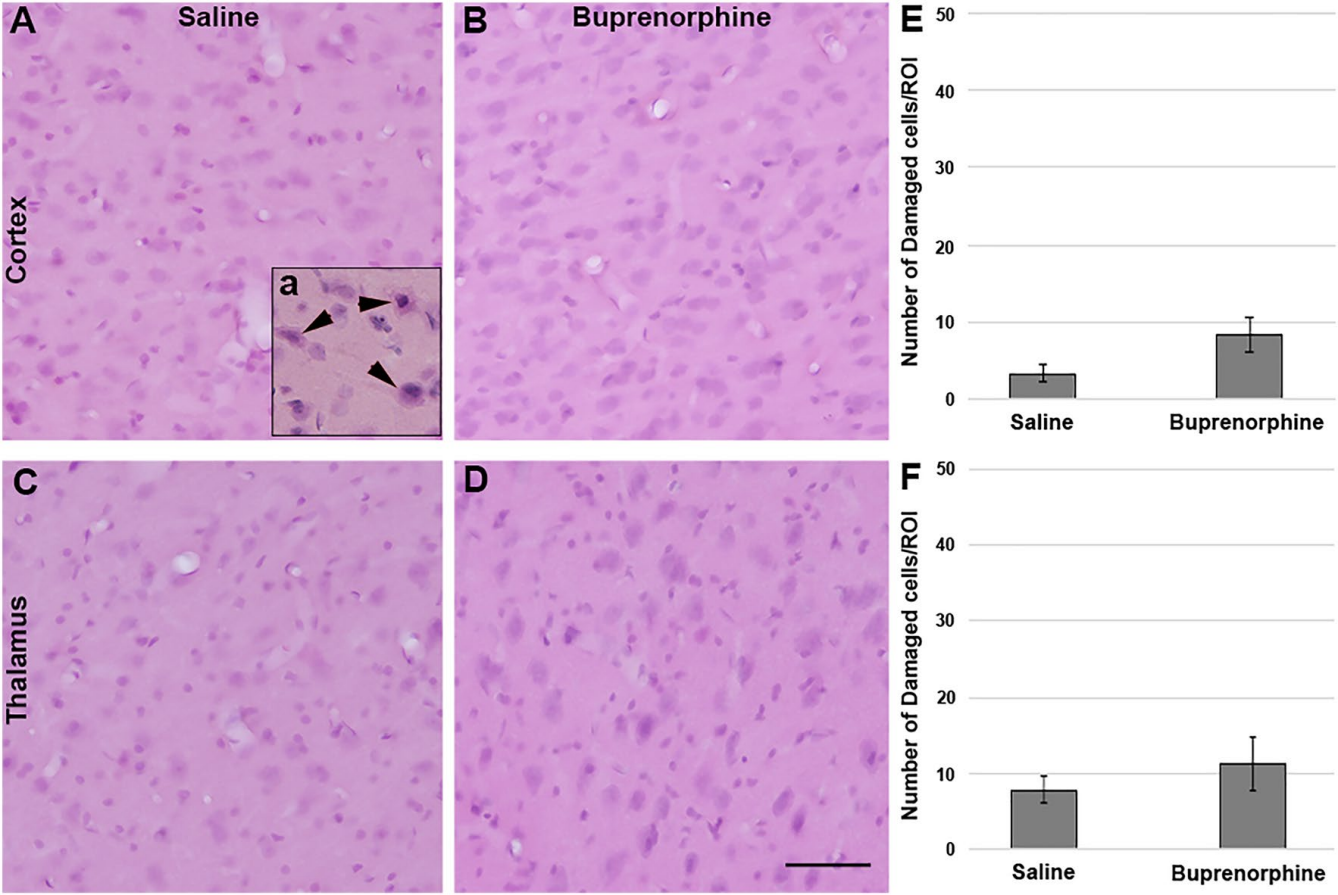
Cell damage was not altered by Bup treatment. Representative photomicrographs from animals treated with (**A**,**C**) saline or (**B**,**D**) buprenorphine following injury and labeled with hematoxylin and eosin (H&E) to assess cell damage and death in (**A**,**B)** cortical and (**C**,**D**) thalamic regions of interest 1d post-injury. Inset **a** is a positive control of damaged cells demonstrating eosinophilic cytoplasm and heterochromatic nuclei. (**E**,**F**) Corresponding bar graphs depicting number of damaged cells per region of interest in (**E**) cortex and (**F**) thalamus. The number of damaged cells was consistent between saline and buprenorphine treated rats in both regions of interest, indicating no change in cell damage or death between treatment groups. Figure was compiled using Adobe Photoshop CS., version 22.0 (2020), San Diego, CA. n = 6 rats/group. Mean ± SEM. Scale = 50 μm.

As CFPI in rats produces significant and various cellular pathology in the lateral neocortex and thalamic domains^25,28^, these regions of interest were the focus of all subsequent pathological assessments. Overt acute cellular damage/death was assessed using H&E staining. Any cell that demonstrated a heterochromatic nucleus and eosinophilic cytoplasm was considered to be damaged and/or undergoing early stages of cell death^26,28,29^ (Fig. 2A). There were very few damaged/dead cells in either the cortex (Fig. 2A–C; One-way ANOVA, F_1,10_ = 0.51, *p* = 0.493) or the thalamus (Fig. 2D–F; One-way ANOVA F_1,10_ = 3.18, *p* = 0.105) of either saline or Bup treated animals and no significant difference between groups was detected for either ROI.

**Figure 3:**
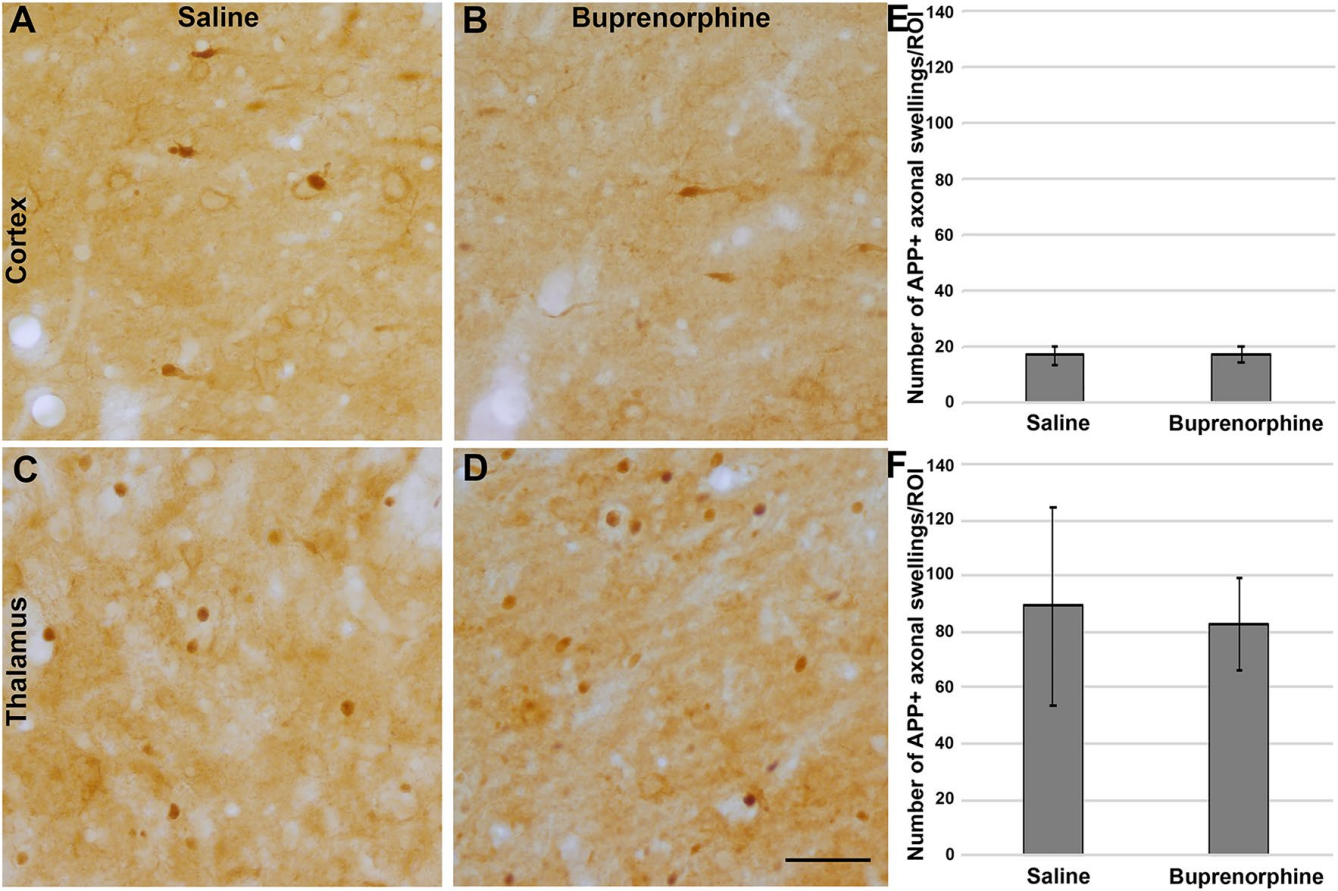
Buprenorphine did not alter acute diffuse axonal injury in either the thalamus or cortex. Representative photomicrographs of (**A**,**C**) saline or (**B**,**D**) buprenorphine treated Sprague Dawley rats 1d following cFPI. Tissue samples of (**A**,**B**) somatosensory neocortex and (**C**,**D**) hemithalamus were labeled immunohistochemically with a primary antibody against amyloid precursor protein (APP) and a secondary biotinylated antibody followed by DAB reaction. Swellings were visualized and quantified to assess axonal injury in each brain region at 1d following cFPI and either saline or buprenorphine treatment. (**E**,**F**) Corresponding bar graphs depicting the number of swellings per region of interest in the (**E**) cortex and (**F**) thalamus. The number of swellings was consistent between saline and buprenorphine treated animals, indicating that buprenorphine does not affect axonal injury in observed regions following TBI. Figure was compiled using Adobe Photoshop CS., version 22.0 (2020), San Diego, CA. n = 6 rats/group; Mean ± SEM. scale = 50 μm.

Diffuse axonal injury (DAI) is a hallmark of mild and/or diffuse TBI and is the leading pathological indicator of injury magnitude following a brain injury^32–34^. Therefore, the total number of amyloid precursor protein positive (APP +) axonal swellings, indicative of DAI-mediated axonal transport dysfunction, was assessed in both the lateral cortex and the thalamus 1d following CFPI and either saline or Bup treatment. While notable DAI was visible in both saline treated (cortex = 17.10 ± 3.39, Thalamus = 89.33 ± 35.50 APP + swelling/ROI) and Bup treated (cortex = 17.15 ± 3.03, Thalamus = 82.71 ± 16.79 APP + swelling/ROI) animals, the degree of DAI was indistinguishable between the two groups in either the cortex (Fig. 3A–C; F_1,10_ = 1.4 × 10^–4^, *p* = 0.990) or the thalamus (Fig. 3D–F; One-way ANOVA F_1,10_ = 0.028, *p* = 0.869).

**Figure 4:**
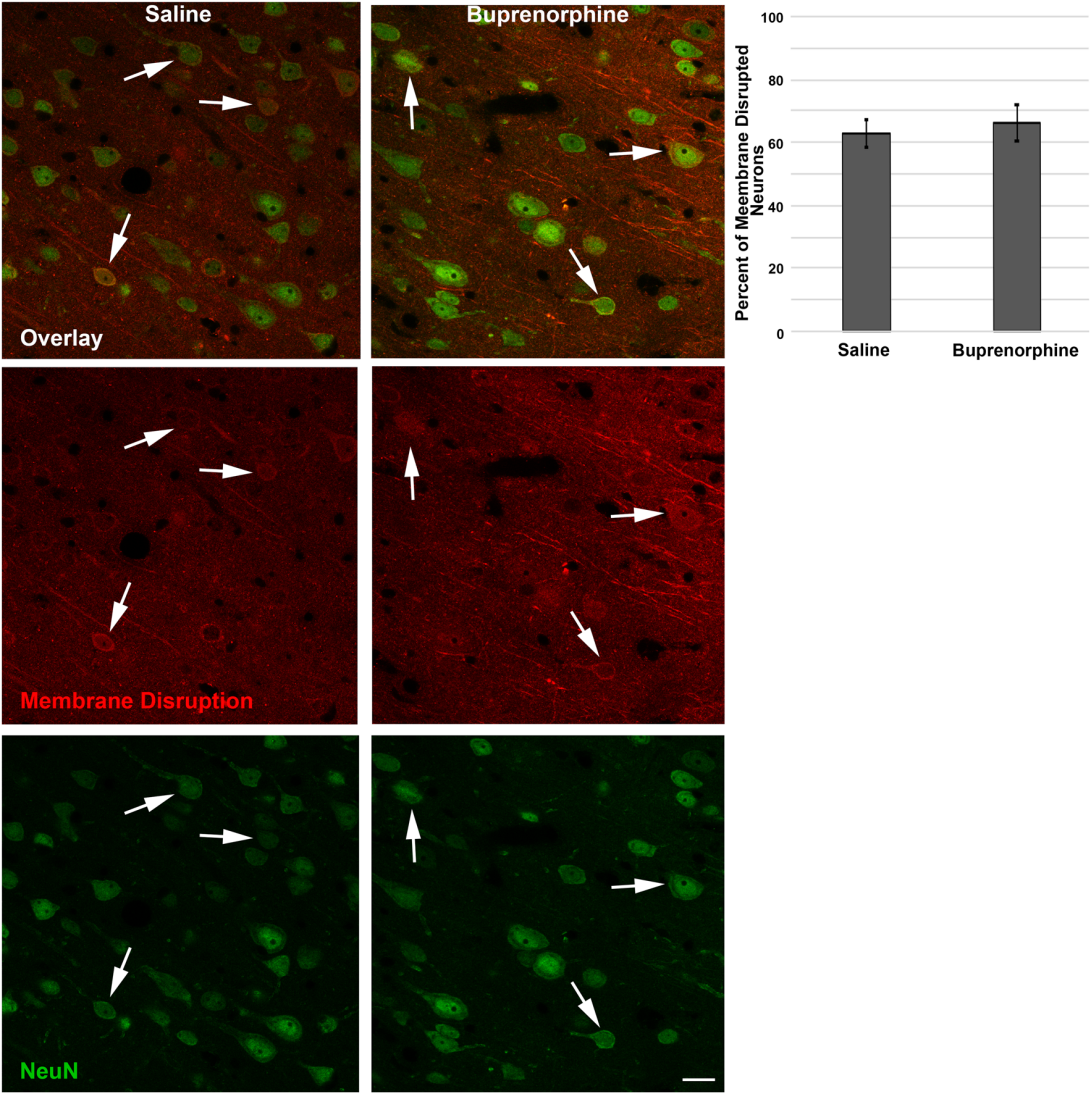
Acute neuronal membrane disruption was not altered in the cortex of Bup-treated animals. Representative photomicrographs of sub-acute neuronal membrane disruption in the lateral neocortex of rats treated with saline or buprenorphine. Prior to sacrifice at 1d post-injury rats were infused with a 10 kDa cell-impermeable, biotinylated dextran. NeuN + neurons are green (single channel images in bottom images) and dextran-containing membrane disrupted neurons are pseudocolored red (single channel images in middle images). Brain sections were incubated with goat anti-biotin primary antibody followed by Alexa-568-conjugated donkey anti-goat secondary antibodies to visualized cells that contained dextrans for the assessment of neuronal membrane disruption (arrows) in the lateral neocortex layers V and VI. Corresponding bar graph depicting the percentage of total neurons that showed disruption in saline and buprenorphine treated animals. The percentage of dextran + neurons was consistent between groups, indicating that buprenorphine treatment did not affect the percentage of membrane disrupted neurons in the cortex. Figure was compiled using Adobe Photoshop CS., version 22.0 (2020), San Diego, CA. n = 6 rats/group. Mean ± SEM. Scale = 20 μm.

Finally, as somatic neuronal damage, in the form of neuronal membrane disruption, is present acutely following both diffuse and focal TBI, and as this pathology is exacerbated by secondary insults, we investigated the potential effects of Bup on neuronal membrane disruption^25,26,29,35–37^. As we and others have demonstrated previously, tagged 10 kDa dextran, which is normally excluded from intact membranes, are a reliable way to evaluate neuronal plasmalemmal disruptions^25,26,29,36,38,39^. However, this method only allows assessment of the cortex, due to the variability of dextran diffusion within the thalamic domain. As found with axonal injury, there was no difference between saline treated and Bup treated groups in regard to the percent of neurons demonstrating membrane disruption 1d following CFPI (Fig. 4; One-way ANOVA F_1,10_ = 0.192, *p* = 0.671).

### Buprenorphine treatment alters microglia and astrocytes in a region-specific manor acutely following diffuse TBI

To assess the potential effects of Bup on acute inflammatory responses in the brain cytokine protein levels were assessed for each ROI in saline and Bup treated animals at 1d post-CFPI. Levels of IL-1a, IL-2, IL-6, IFNy, and TNFα were consistently below detectable limits for nearly all samples tested regardless of treatment group. Concentration of IL-1b, IL-4, IL-10, and IL-12, however, were measurable for the majority of samples tested. While there was no discernable treatment effect for any of the cytokines evaluated in either region (Two-way ANOVA; Treatment IL-1b F_1,16_ = 0.08 *p* = 0.787; IL-4 F_1,16_ = 0.63 *p* = 0.439; IL-10 F_1,16_ = 0.312 *p* = 0.584; IL-12 F_1,16_ = 0.619 *p* = 0.443), there did appear to be a regional difference in expression of IL-1b (One-way ANOVA: F_3,16_ = 7.592, *p* = 0.002; Two-way ANOVA: Region F_1,16_ = 22.53, *p* < 0.001), IL-4 (One-way ANOVA: F_3,16_ = 13.208, *p* < 0.001; Two-way ANOVA; Region F_1,16_ = 38.53, *p* < 0.001), and IL-10 (One-way ANOVA: F_3,16_ = 12.029, *p* < 0.001; Two-way ANOVA; Region F_1,16_ = 35.75 *p* < 0.001) with consistently more cytokine expression in the cortex as compared to the thalamus (Table 2).

**Table 2:** Cytokine concentrations in cortex and thalamus of saline and buprenorphine treated animals at 1d following CFPI.

	Cortex		Thalamus	
	Saline	Buprenorphine	Saline	Buprenorphine
IL-1b pg/mg	14.40 (4.44)	14.40 (11.45)	0.36 (0.50) #	2.29 (2.29) #
IL-4 pg/mg	12.78 (6.23)	12.97 (2.51)	1.13 (1.15) #	3.63 (3.29) #
IL-10 pg/mg	17.12 (7.71)	18.69 (3.79)	4.36 (2.77) #	5.23 (3.83) #
IL-12 pg/mg	36.93 (23.04)	33.41 (28.87)	19.64 (43.93)	2.84 (6.35)

n = 6 rats/group. Data presented as mean (SD) pg of cytokine per mg of total protein. Regional differences: #*p* < 0.05 compared to cortex.

**Figure 5:**
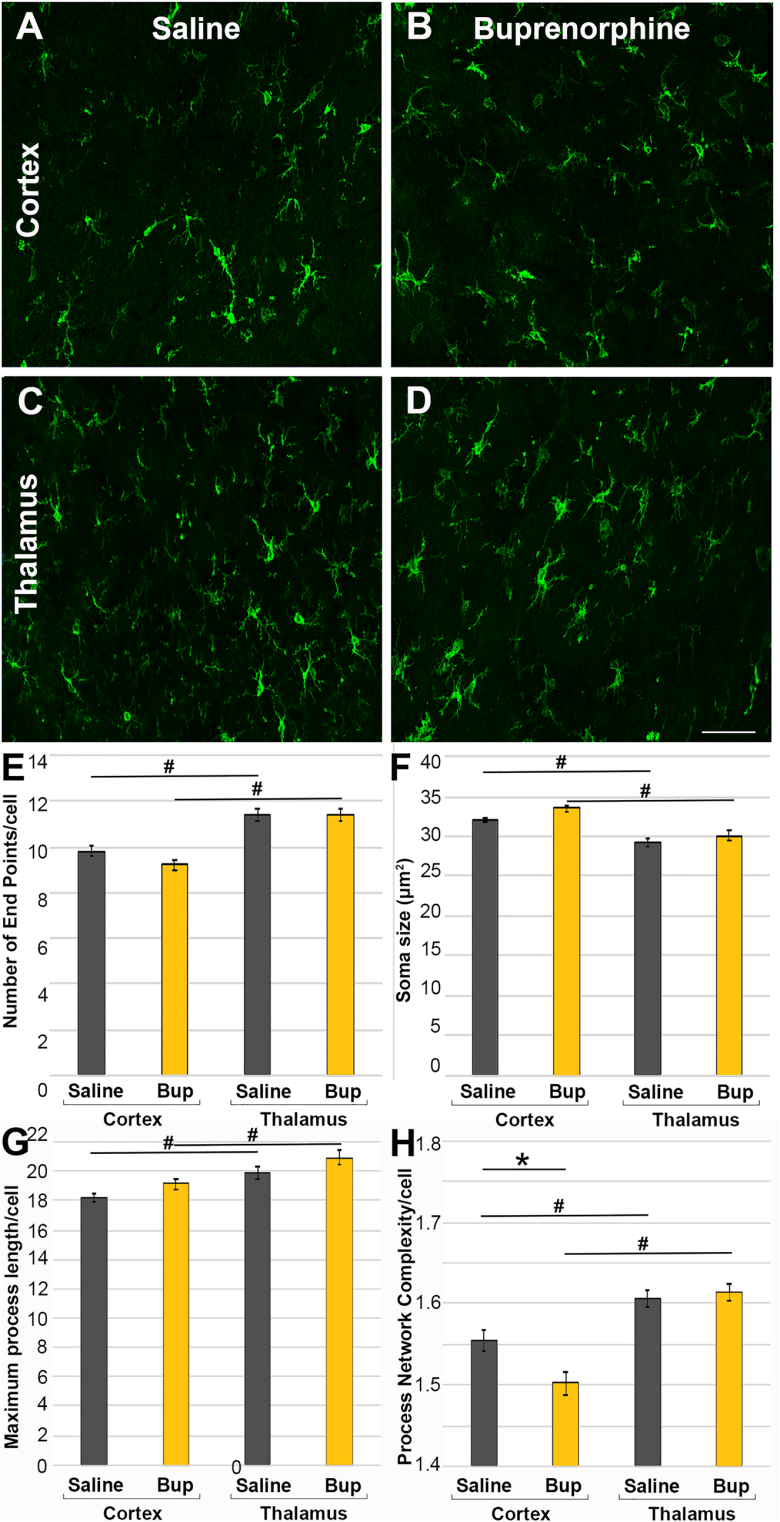
Microglia demonstrate a more active morphology in the cortex, but not the thalamus, following Bup-SR-Lab treatment. Representative photomicrographs of (**A**,**B**) cortex and (**B**,**C**) thalamus from rats 1d post-cFPI and treated with (**A**,**C**) saline or (**B**,**D**) buprenorphine (Bup). The microglial calcium binding protein, Iba-1, is labeled in green. Bar graphs depicting microglial morphological characteristics indicative of activation; (**E**) number of process end points/cell, (**F**) average soma size, (**G**) average maximum process length and (**H**) the average complexity of the microglial process network/cell. Microglial morphologies were significantly different in the cortex compared to microglia found within the thalamus, regardless of treatment. Additionally, microglial somal size and process network complexity was altered in the cortex, but not the thalamus of Bup-treated rats, suggesting regional specificity in the effects of Bup. Figure was compiled using Adobe Photoshop CS., version 22.0 (2020), San Diego, CA. Treatment differences: *p < 0.05 compared to saline; regional differences: #*p* < 0.05 compared to cortex. n = 6 rats/group. Mean ± SEM. Scale = 50 μm.

To further investigate the potential that more subtle changes in neuroinflammation were occurring acutely following Bup treatment, microglia and astrocyte morphologies were analyzed. Alterations in microglial morphology indicative of an altered activation state (larger soma size, fewer process endpoints, shorter processes, and reductions in overall cell complexity^30,40,41^) were assessed at 1d following CFPI in the cortex and thalamus of animals treated with saline or Bup. Microglia within the cortex of saline treated animals demonstrated 9.80 ± 0.21 process endpoints/cell, with somal perimeters of 32.11 ± 0.28 µm^2^ and a maximum process segment length of 18.21 ± 0.28 µm as well as an average cell complexity index of 1.56 ± 0.01 arbitrary units. Cortical microglia demonstrated alterations in Bup treated animals as compared to saline. Cortical microglia in Bup treated animals also demonstrated approximately 9 endpoints/cell (9.24 ± 0.22) and a maximum process segment length of 19.14 µm ± 0.31 but showed increased somal sizes (33.48 ± 0.3 µm^2^ One-way-ANOVA, F_3,2627_ = 19.2, *p* < 0.001; Two-way ANOVA Treatment, F_1,2627_ = 5.97, *p* = 0.015) and reduced process network complexity/microglia (1.50 ± 0.01 µm^2^ One-way-ANOVA F_3,2627_ = 17.85, *p* < 0.001; Two-way ANOVA Treatment, F_1,2627_ = 3.253, *p* = 0.07) compared to saline treated cortical microglia (Fig. 5). There was a significant interaction between drug treatment and region for the microglial complexity index (Two-way-ANOVA Region*Treatment F_1,2627_ = 6.15, *p* = 0.013) but not for any other metric. Microglia within the thalamic domain, however, demonstrated approximately 11 process endpoints/cell (saline = 11.38 ± 0.25; Bup = 11.39 ± 0.25), with somal perimeters of about 30 µm^2^ (saline = 29.20 ± 0.49 µm^2^; Bup = 30.01 ± 0.64 µm^2^) and a maximum process segment length of around 20 µm (saline = 19.83 ± 0.42 µm; Bup = 20.95 ± 0.50 µm) as well as an average cell complexity index of ~ 1.6 (saline = 1.606 ± 0.01; Bup = 1.614 ± 0.01 arbitrary units). There were no significant differences in any of these metrics between thalamic microglia in saline vs. Bup treated groups (Fig. 5). All microglial morphological metrics demonstrated significant region-specific differences in which the thalamus displayed increased numbers of process endpoints (Two-way-ANOVA; Region F_1,2627_ = 64.89, *p* < 0.001), smaller soma sizes (Two-way-ANOVA; Region F_1,2627_ = 51.10, *p* < 0.001), longer maximum process length/cell (Two-way-ANOVA; Region F_1,2627_ = 19.67, *p* < 0.001), and increased process network complexity (Two-way-ANOVA; Region F_1,2627_ = 44.05, *p* < 0.001) per microglia compared to the cortex in either saline or Bup-treated animals (Fig. 5).

**Figure 6:**
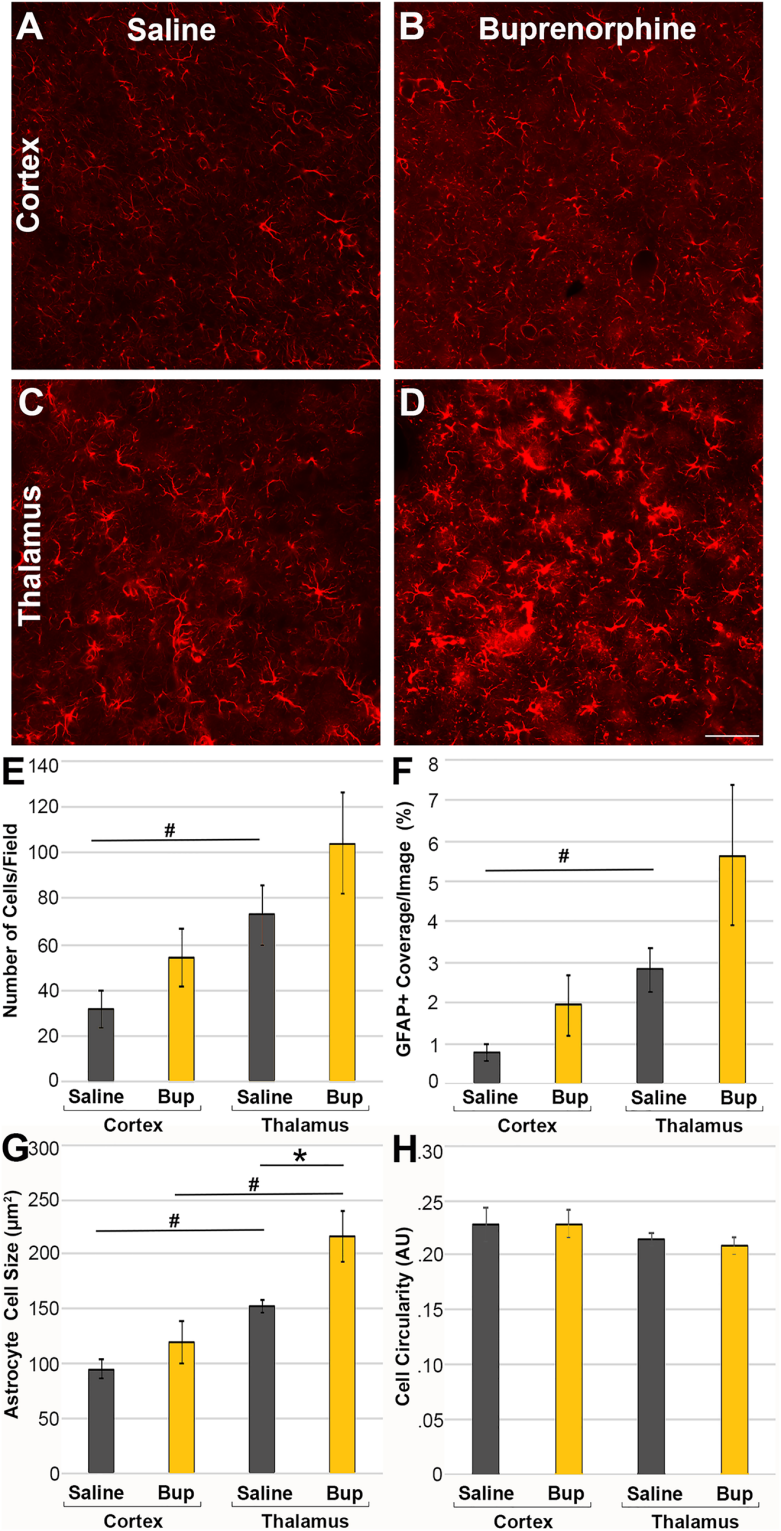
Astrocytes are larger with Bup-treatment in the thalamus, but not in the cortex. Representative photomicrographs of the astrocytic glial fibrillary acidic protein (GFAP) in (**A**,**B**) cortex and (**C**,**D**) thalamus from rats 1d post-cFPI and treated with (**A**,**C**) saline or (**B**,**D**) buprenorphine (Bup). Bar graphs depicting astrocyte (**E**) cell number, (**F**) % of GFAP + coverage/image, (**G**) average astrocyte cell size, and (**H**) astrocyte cell circularity. There were more, larger GFAP + astrocytes covering a greater area in the thalamus compared to the cortex of saline treated rats 1d following cFPI. Thalamic astrocytes were larger with Bup treatment compared to those in saline treated rats, however cortical astrocyte size was not significantly altered by Bup treatment. Figure was compiled using Adobe Photoshop CS., version 22.0 (2020), San Diego, CA. Treatment differences: *p < 0.05 compared to saline; regional differences: #*p* < 0.05 compared to cortex. n = 6 rats/group. Mean ± SEM. Scale = 50 μm.

As opposed to microglia, cortical GFAP + astrocytes, did not appear significantly altered 1d following TBI and Bup treatment as compared to saline treated animals (Fig. 6). There were trends toward higher numbers of astrocytes/image (One-way-ANOVA; F_3,16_ = 4.3, *p* = 0.02; Two-way-ANOVA; Treatment F_1,16_ = 3.31, *p* = 0.09) covering a greater percentage of the image (One-way-ANOVA F_3,16_ = 4.42, *p* = 0.02; Two-way-ANOVA; Treatment F_1,16_ = 4.11, *p* = 0.06) between saline and Bup treatment in the cortex, however, neither of these trends were statistically significant. While astrocyte cell size was consistent between saline and Bup treatment groups in the cortex, thalamic astrocytes demonstrated greater cell size in the Bup-treated animals compared to the saline treated animals at 1d post-injury (One-way-ANOVA; F_3,16_ = 11.26, *p* < 0.001; Two-way-ANOVA; Treatment F_1,16_ = 8.12, *p* = 0.01; Fig. 6G). There were also significant regional differences in the morphology of astrocytes in the control animals, with more astrocytes (Two-way-ANOVA; Region F = _1,16_ = 9.49, *p* = 0.007) covering a larger proportion of the image (Two-way-ANOVA; Region F = _1,16_ = 8.43, *p* = 0.01) in the thalamus of saline-treated control animals compared to the cortical astrocytes. These regional differences were not maintained following Bup treatment (Fig. 6). The size of astrocytes, however, was (Two-way-ANOVA; Region F = _1,16_ = 24.04, *p* < 0.001; Fig. 6G). The circularity of astrocytes was also consistent between treatment groups and ROIs (One-way-ANOVA; F_3,16_ = 0.85, *p* = 0.49; Fig. 6H). There were no interactions between region and treatment for any metrics.

### Treatment with Buprenorphine altered myelin basic protein (MBP) isoforms but not overall MBP expression

**Figure 7:**
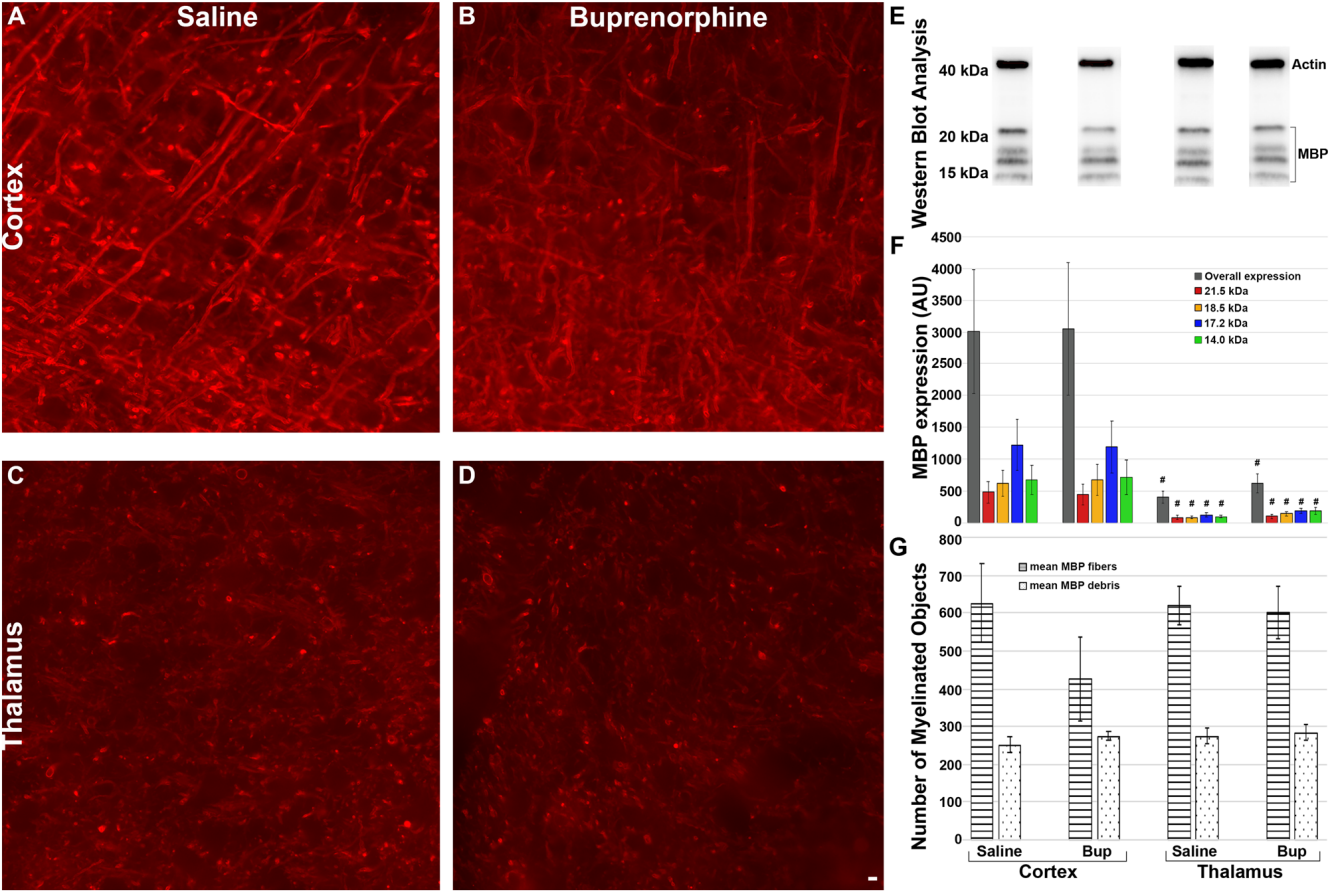
Acute myelin debris and MBP expression was unchanged with Bup treatment but was different between cortex and thalamus. Representative photomicrographs of myelin integrity in (**A**,**B**) cortex treated with saline or buprenorphine (Bup) and (**C**,**D**) thalamus treated with saline or Bup, analyzed using ImageJ. Representative bands for (**E**) Western blot analysis of actin protein (band at ~ 40 kDa) and myelin basic protein (MBP; bands at ~ 15–20 kDa). (**F**) Bar graph depicting average overall MBP expression (gray bars), as well as expression of 21.5 kDa (red bars), 18.5 kDa (orange bars), 17.2 kDa (blue bars), and 14.0 kDa (green bars) MBP isoforms in the cortex and thalamus of saline and Bup treated animals. Overall MBP expression and expression of individual MBP isoforms were not significant in either saline or Bup treatment groups. There were, however, significant regional differences with higher MBP expression in the cortex than in the thalamus. (**G**) Bar graph depicting average number of MBP fibers and MBP debris of cortex and thalamus in saline and Bup treated animals. The number of myelinated fibers was consistent between groups, indicating neither region was affected by buprenorphine treatment. Figure was compiled using Adobe Photoshop CS., version 22.0 (2020), San Diego, CA. Treatment differences: regional differences: #*p* < 0.05 compared to cortex. n = 6 rats/group. Mean ± SEM. Scale = 50 μm.

In order to assess the effect of Bup on myelin pathology at 1d post-CFPI, immunohistochemical labeling (Fig. 7A–D) and Western blotting were performed to evaluate myelin integrity and the expression of myelin basic protein (MBP). There was no significant difference in the total number of intact myelin fibers (One-way-ANOVA F_3,12_ = 1.224, *p* = 0.344) or myelin debris (One-way-ANOVA F_3,12_ = 0.558, *p* = 0.653) between ROI or drug treatment groups (Fig. 7E). Furthermore, overall MBP expression did not appear to be significantly altered with Bup treatment in either region (Two-way-ANOVA Treatment F_1,16_ = 0.064, *p* = 0.803). There did appear to be statistically significant regional differences, however, where overall expression of MBP was higher in the cortex than the thalamus, regardless of drug treatment group (One-way ANOVA F_3,16_ = 11.22, *p* < 0.001; Two-way-ANOVA; Region F_1,16_ = 33.576, *p* < 0.001). As the different MBP isoforms are linked to different developmental stages of myelination^42^, the individual MBP isoforms (21.5 kDa, 18.5 kDa, 17.2 kDa, and 14.0 kDa) were also analyzed. Similar to analysis of the overall expression of MBP, the expression of individual MBP isoforms was not statistically different between Bup and saline treatment groups (Two-way ANOVA; Treatment 21.5 kDa: F_1,16_ = 0.054, *p* = 0.819, 18.5 kDa: F_1,16_ = 0.127, *p* = 0.727, 17.2 kDa: F_1,16_ = 0.001, *p* = 0.982, and 14.0 kDa: F_1,16_ = 1.85, *p* = 0.673; Fig. 7G). There were, however, statistically significant regional differences for all isoforms (One-way ANOVA; 21.5 kDa: F_3,16_ = 12.410, *p* < 0.001, 18.5 kDa: F_3,16_ = 6.363, *p* = 0.005, 17.2 kDa: F_3,16_ = 17.202, *p* < 0.001, and 14.0 kDa: F_3,16_ = 5.364, *p* = 0.01) in which the expressions of all isoforms were higher in the cortex of either saline or Bup treatment group compared to the thalamus (Two-way ANOVA Region 21.5 kDa F_1,16_ = 37.1, *p* < 0.001; 18.5 kDa F_1,16_ = 19.0, *p* < 0.001; 17.2 kDa F_1,16_ = 51.6, *p* < 0.001; 14.0 kDa F_1,16_ = 15.9, *p* < 0.001; Fig. 7G).

## Discussion

Buprenorphine (Bup) is a semi-synthetic opioid which is a long-lasting and very effective partial agonist of the Mu opioid receptor (MOR) and an antagonist of the Kappa opioid receptor (KOR) and at higher doses the delta opioid receptor (DOR)^10,12^. The efficacy of low dose Bup in blocking the agonist effects of other opioids while also reducing withdrawal symptoms has made it a primary treatment for opioid use disorder^14^. Additionally, as there is a highly effective slow-release formula of Bup, that effectively alleviates pain for multiple days following a single subcutaneous injection, Bup is commonly used in experimental studies as a post-operative analgesic^16,19–21^. To our knowledge there have been very few studies evaluating the potential effects of Bup following CNS injury. One study found that Bup significantly reduced distress after subarachnoid hemorrhage but not controlled cortical impact injury and failed to alter pain and stress levels after TBI following administration of 0.1 mg/kg Bup during anesthesia before surgery and another dose repeated after 12h^43^. In another study, no differences were found in focal pathology following spinal cord injury^42^. However, there have been no studies systematically assessing the potential effects of Bup-SR-Lab on the acute diffuse pathology produced by CNS injury. As the potential for analgesic-induced alterations on pathological progression give some investigators pause, the current study evaluated the effects of Bup-SR-Lab on acute physiological and diffuse pathological changes following TBI.

Although in previous studies Bup demonstrated physiological effects, such as respiratory depression and bradycardia^44^, we found that subcutaneous treatment with 1 mg/kg Bup 15 min post-injury appeared to have no effect on systemic physiology. Specifically, heart rate and hemoglobin oxygen saturation were comparable between saline and Bup treated animals (Fig. 1). While all animals in the current study were ventilated to maintain consistent respiratory rates, previous, studies found that Bup could act as a respiratory depressant. One study found that when rats were given a dose of Bup ranging from 1 to 3 mg/kg respiratory rates decreased in a dose-dependent fashion^45^. Another study found that rats treated with 1.4 mg/kg, 4.3 mg/kg or 8.6 mg/kg doses of Bup displayed a ceiling in Bup-induced respiratory depression after nearly 2 h of Bup infusion^46^. In another study, arterial pH and PaCO_2_ were not altered in rats treated with 1.2 mg/kg Bup-SR but did demonstrate lower arterial oxygen saturation compared to saline controls, indicating potential respiratory alterations^19^. This ceiling phenomenon, where respiratory depression reached its apparent maximum effect regardless of drug dose, is potentially attributed to Bup’s partial agonism at the MOR and may have prevented rapid changes in respiratory rate, as well as changes involving neural systems and behavioral processes^47^.

While all animals lost weight following TBI, animals treated with Bup demonstrated better maintenance of their body weight within the first day following CFPI compared to the saline control group (Fig. 1). In a previous study using Bup-SR, weight was measured within the first 4 days after 0.3–4.5 mg/kg doses were given to rats. Rats that were given 1.2 mg/kg doses of Bup-SR maintained their weight but rats given 0.3 mg/kg and 4.5 mg/kg of Bup-SR gained or lost weight, respectively^16^. This indicates that there may be a dose-regulated response to Bup in regard to weight change in rats. Another study examining rabbits found that treatment of 0.03 mg/kg of Bup every 12 h for 48 h 1d post-operatively experienced immediate weight loss post-operatively that sustained over the next four days, however, the rats displayed a slow return to baseline body weight by day 5 post-operatively^48^*.* The reduced weight loss we observed at 1 day-post-injury may be attributed to the effects of Bup on gastrointestinal motility rather than suppressed appetite^49^. In a study examining the effects of 0.05 mg/kg buprenorphine every 12 h for 48 h on rabbits found that buprenorphine induced gastrointestinal hypomotility of gut and delayed the passage of feces. Wheel running activity alters not only energy but food intake, neural systems involving stress response, and behavioral processes. A study found that voluntary wheel running activity was significantly lower at 24 h with Bup treatment but returned to baseline by 48 h^19^, which could impact body weight.

The weight retention could also be mediated by the KOR. Kappa-opioid activation is known to cause stress, which is highly linked to weight loss^50^, but Bup acts as an antagonist of the KOR, which could reduce the effect of KOR-mediated weight loss. However, we were unable to find any studies investigating the association between KOR-activation and weight changes. Finally, while our study did not inspect the metabolism effects of Bup, it is known that Bup partially metabolizes to norbuprenorphine in the liver and both compounds are excreted as glucuronides. They then undergo enterohepatic circulation, where the drug and its metabolites can remain in intestinal circulation for days^51^. One study investigating Bup glucuronide metabolites discovered that two glucuronide metabolites of Bup are pharmacologically active^52^. This suggests that even as Bup is broken down in order to be eliminated from the body, its metabolites are still functioning and could have effects on weight loss at longer time points than the current study investigated.

While we did not observe any remarkable concerns regarding weight loss in animals receiving Bup-SR-Lab at 1d following cFPI, there have been observations linking different formularies of Bup with pica in rats. As this was an acute 1d study, we did not assess longer term weight changes in rats with Bup-SR-Lab. Additionally, there was no sham group in the current study so it is unknown what the combined effects of TBI and Bup-SR-Lab are in rats but we are currently in the process of investigating longer term studies where weight loss may be exacerbated due to injury as well as Bup-SR-Lab as compared to sham injured groups.

Although both treatment groups presented DAI, there were no discernable differences in axonal injury between groups, indicated by the total number of APP + axonal swellings in either the thalamus or cortex. There were also no significant indications of cell damage/death 1d post-CFPI in either region regardless of Bup treatment. Contrarily, a previous study found that Bup administration prevented neuronal death and protected neurons in the medial and lateral regions of the thalamic reticular nucleus following resuscitation from cardiac arrest^53^. This region-specific finding was interesting as MORs are present in the medial and lateral regions of the thalamic reticular nucleus but not in the central regions^53^. This suggests that Bup may selectively protect regions in a MOR-mediated fashion. As the CFPI model used in our study does not precipitate cell death post-injury in either the cortex or the thalamus^28,29,54,55^, it is unsurprising that Bup’s potential neuro-protective effects were not seen in the current study.

Acute neuroinflammation, however, does occur following CFPI as it does in human TBI^30,55–60^. The primary cellular representatives for neuroinflammatory changes are microglia and astrocyte activations. The neuroinflammatory response of activated microglia and astrocytes occurs on a spectrum ranging from a pro-inflammatory phenotype, which is characterized by neurotoxic properties and release of neuroinflammatory cytokines, to an anti-inflammatory phenotype, which is characterized by the release of neurotrophic factors and anti-inflammatory cytokines that promote repair^61^. This correlates with the findings of higher cytokine expression (IL-1b, IL-4, IL-10, and IL-12) in the cortex, but our data found no discernable differences between treatment groups within either brain region analyzed. Corroboratively, studies have shown that animals treated with Bup, do not display systemic immunosuppression, which is common for MOR agonists, such as fentanyl^62^. One study, however, found higher levels of cytokines, such as IL-10 and TNFα, in the serum of animals treated with Bup compared to the control animals^63^, suggesting a potentially unique role of Bup on neuroinflammation as compared to other opioids. Another study recently found increased expression of IL-1b in the hippocampus of aged rats treated with morphine^64^ weeks following surgical procedures, indicating that there might also be neuroinflammatory changes at later time points, which this study would not have captured. These possibilities will need to be further assessed at later time points following diffuse TBI, when neuroinflammatory responses are more robust^40,56^.

Microglial and astrocyte activation can also be assessed via investigation of the morphological remodeling each cell type undergoes. Activated microglia demonstrate larger soma size, fewer process endpoints, shorter processes, and reductions in overall cell complexity as compared to non-activated ramified surveying microglia^30,41,55,65^. Activated astrocytes undergo hypertrophy and increased expression of the intermediate filament, GFAP^66,67^. Such activation-associated morphological changes of both microglia and astrocytes are seen in various brain regions following diffuse TBI^30,40,41,66,68,69^, however, the effects of Bup on such changes are not well understood. Both microglia and astrocytes express all three opioid receptors, suggesting that Bup could potentially play a role in modulation of microglial and/or astrocyte morphological changes associated with activation^10^. Our current studies found that Bup treatment was linked to a more activated morphological phenotype of microglia. Previous studies found that opioids given during development drastically reduce microglial process branching and hence the overall size and complexity of microglia; morphological changes indicative of microglial activation^9^. Other studies found that microglial activation is increased in a model of chronic morphine administration but can be reduced by the non-specific opioid receptor antagonist, Naloxone^70,71^. The effect of Bup on the immune system is not fully understood, but there is likely an interplay between its roles as a partial agonist at the MOR and antagonistic properties toward the other opioid receptors.

Our findings also indicate potential regional specificity of Bup-associated microglial activation. When comparing the microglial morphology between the two regions in both saline and Bup-treated animals, cortical microglia were found to exhibited larger soma, reduced process network complexity, shorter maximum process length/cell, and decreased numbers of process endpoints per microglia than the thalamus, regardless of treatment group. Additionally, microglia in the cortex demonstrated morphological changes associated with activation (larger cell bodies and reduced process network complexities), whereas these Bup-associated microglial changes were not observed in the thalamus.

Alternatively, Bup-treated rats demonstrated an increase in astrocyte cell size, indicative of activation-linked hypertrophy, in the thalamus that was not discernable in the cortex of Bup-treated rats. Previous studies have found that astrocyte signaling is affected by buprenorphine. Treatment with Bup was associated with an upregulation of GFAP intensity^72^. Another study, however, found that Bup treatment reduced GFAP intensity in a model of morphine-induced dependence, demonstrating that astrocyte activation could be impacted by the interplay of multiple opioids^73^. Because Bup is a partial agonist of the MOR, at higher doses, Bup could induce an inhibition of the MOR that could impact microglial and astrocyte activation differentially^74^. The MOR is more highly expressed in the thalamus as compared to the lateral neocortex, which has low to moderate opioid receptor expression^75^. Therefore, the regional-specific differences between Bup effects on microglial and astrocyte morphologies could be linked to differences in the opioid receptor profiles between these two brain regions.

A previous study found that Bup exposure during development had a significant impact on myelin protein expression. Specifically, it was found that a low dose of 0.3 mg/kg Bup given to early postnatal rat pups resulted in significantly increased expression of all MBP isoforms^23^. However, when given at a higher dose of 1 mg/kg, expression of MBP was delayed^74^. While our study utilized a dose of 1 mg/kg Bup-SR-Lab, we found no change in overall MBP expression between the Bup treatment group and the saline treatment group. We did, however, observe regional differences between the cortex and thalamus, with higher expression of MBP in the cortex than in the thalamus (Fig. 7F). This could be due to the adult age at which our study was conducted, in which developmental myelination has already occurred and delays in MBP expression upon injury and subsequent repair might be more subtle.

The myelin protein, MBP, consists of four major isoforms with molecular weights of 21.5 kDa, 18.5 kDa, 17.2 kDa, and 14.0 kDa, produced via alternate splicing of the primary MBP transcript^42^. One study investigating the degradation of MBP following TBI reported that all four major isoforms of MBP were degraded within hours following injury and intact protein levels did not return to base levels for 3–5 days post-injury^76^. This study observed significant proteolysis of MBP in the cortex hours post-injury but found MBP breakdown in the hippocampus was more delayed with MBP breakdown not peaking until 48 h after TBI potentially due to differences in the compression-induced contusion force^76^. It is possible that in the current study, we observed higher levels of MBP expression in the cortex as compared to the thalamus due to such differences in biomechanical forces, however, in this study we were assessing MBP and not the breakdown of MBP. It is also possible that the observed difference in MBP expression in the cortex vs. the thalamus is reflective of the proportion of myelinated fibers within each region. Ultimately, it is likely that myelin pathology alterations could evolve over longer post-injury time-points following CFPI.

## Conclusions

The findings of this initial study show that preclinical use of Bup-SR-Lab has little effect on acute pathology following diffuse brain injury. However, because only one acute time point was observed in this study, future studies are required to examine potential long-term differences in physiological and pathological outcomes following TBI. Additionally, as morphological aspects of both microglia and astrocytes were found to be changed by Bup-SR-Lab treatment, metanalytical studies assessing these parameters should be aware of the potential effects of post-injury Bup treatment on these outcome metrics. These subtle changes, however, would not preclude the use of Bup-SR-Lab for post-TBI pain management in primary acute-survival animal studies.

## Supplementary information

Supplementary Figure 1.

## Data Availability

The datasets used and/or analyzed during the current study are available from the corresponding author on reasonable request.

## Abbreviations

TBI: Traumatic brain injury
Bup-SR-Lab: Buprenorphine-Slow-release-Lab
CFPI: Central fluid percussion injury
ROI: Region of interest
MBP: Myelin basic protein
DAI: Diffuse axonal injury
FITBIR: Federal Interagency Traumatic Brain Injury Research
Bup: Buprenorphine
H&E: Hematoxylin and eosin
Cat: Catalogue
SEM: Standard error of the mean
APP: Amyloid precursor protein
MOR: Mu opioid receptor
KOR: Kappa opioid receptor
DOR: Delta opioid receptor
CNS: Central nervous system
GFAP: Glial fibrillary acidic protein
