# Supplementary material for: Buprenorphine alters microglia and astrocytes acutely following diffuse traumatic brain injury
Source: Sci Rep. 2021 Apr 21;11:8620. doi: 10.1038/s41598-021-88030-z (PMC8060410; doi:10.1038/s41598-021-88030-z)
Supplement: Supplementary file 1 — Supplementary Figure 1. [file 41598_2021_88030_MOESM1_ESM.docx]

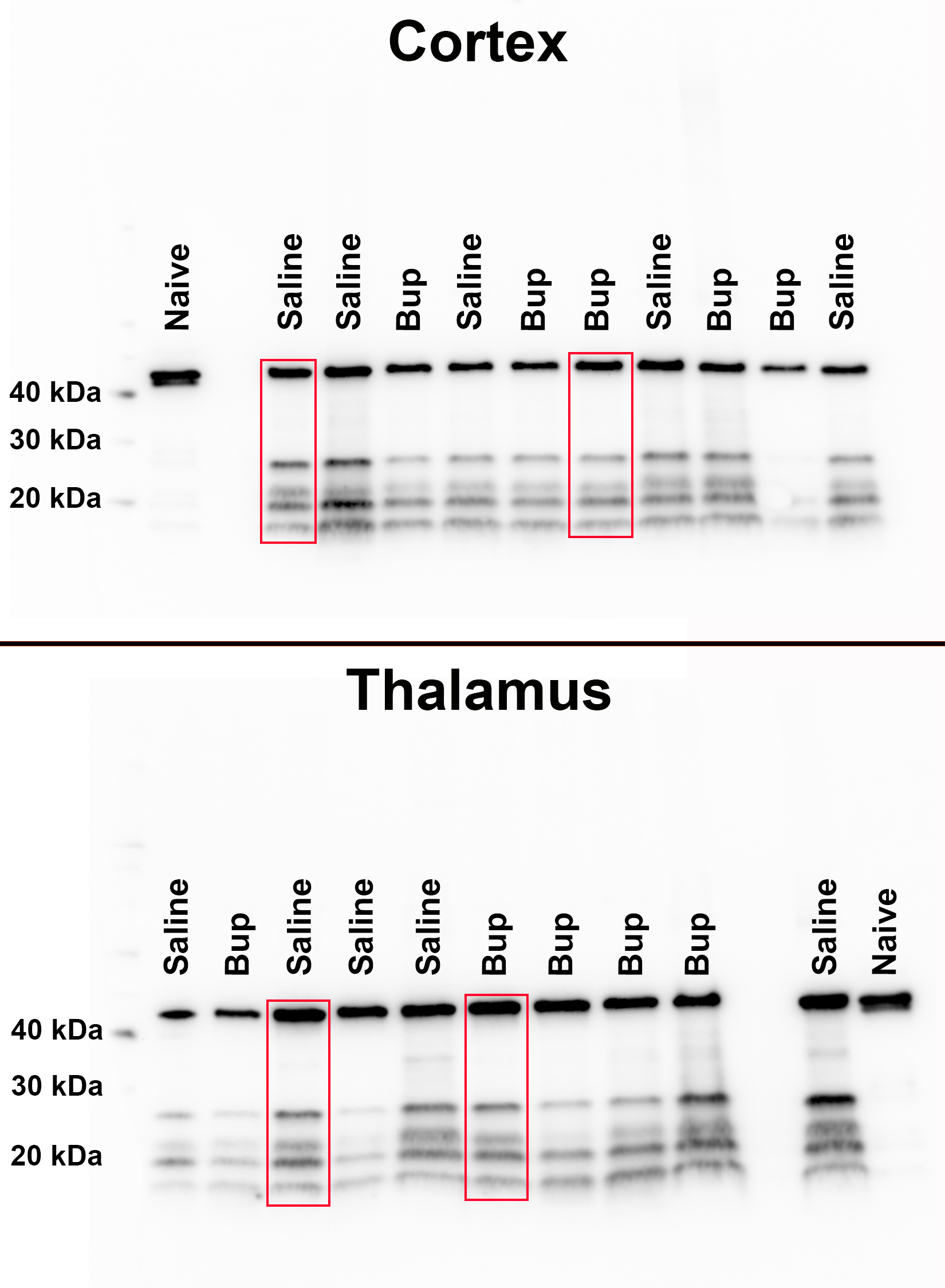


**Supplemental Figure 1.** Representative full membrane western blots of cortical and thalamic samples labeled for actin (band at ~40kDa) and myelin basic protein (MBP; bands at ~15-20 kDa). Representative cropped lanes used for figure 7 are highlighted with red boxes. Each sample was run in independent triplicates and all samples were normalized to the same naïve control sample. Figure was compiled using Adobe Photoshop CS., version 22.0 (2020), San Diego, CA.
